# “It was protected, except, it wasn’t [with] a condom": a mixed-methods study of BBVs/STIs protective practices among International University Students in Sydney, Australia

**DOI:** 10.1186/s12889-022-14512-y

**Published:** 2022-11-24

**Authors:** Sylvester Reuben Okeke

**Affiliations:** Centre for Social Research in Health, UNSW Sydney, Sydney, Australia

**Keywords:** Acculturation, Migration, HIV, Condom, Contraception, Sexual norms

## Abstract

**Background:**

A number of previous sex-related studies among international students in Australia and other Western societies may be limited by conflating students from conservative and non-conservative sexual backgrounds. Such conflation leads to situations where nuances and complexities around sex-related experiences are lost or, at most, tangentially investigated. To address this research problem, this study used a mixed-methods design to examine protective practices against blood-borne viruses and sexually transmissible infections (BBVs/STIs) among Sydney-based East Asian and sub-Saharan African international students.

**Methods:**

This mixed-methods study generated quantitative data using anonymous online survey (*n* = 149), and qualitative data through in-depth interviews (*n* = 20). The main recruitment strategy involved advertising the study through paper and electronic flyers. Quantitative data were analysed using logistic regression, while interviews data were analysed using reflexive thematic analysis.

**Results:**

Self-reported BBVs/STIs protective practices in the last 12 months include abstinence (28.7%), consistent condom use (19.9%), occasional condom use (18.7%), single partner fidelity (25.1%), other strategies (1.8%), and nothing (5.8%). Further, findings from the bivariate analysis showed higher BBVs/STIs prevention knowledge, lower acculturation into Australian sexual culture, greater access to sexual health information, less conservative sexual norms, greater emotional social support and older age were significantly associated with increased protective practices. Variables significant at bivariate level were entered into a logistic regression. The model was statistically significant, (*X*^2^(6) = 31.33, *p <* 0.01) and explained 33.1% of the variance in BBVs/STIs protective practices. However, only acculturation to sexual norms in Australia (OR = 0.883, 95% CI = 0.820–0.952) was found to be independent predictor of BBVs/STIs protective practices.

The results of the study based on the quantitative data, indicated condom use (consistent and occasional) was the most reported BBVs/STIs protective behaviour among sexually active participants. Therefore, interviews data was used to explore condom-use motives and practices. The interviews results showed participants primary concern as regards condom use was around pregnancy and not BBVs/STIs. Thus, some participants described safe sex largely as contraception, with BBVs/STIs constituting a secondary concern or no concern at all.

**Conclusions:**

Based on the results of this study, tailored sexual health interventions for international students; which incorporate strategies for modifying perceived sexual norms in Australia, are advocated. In addition, this study recommends sexual health interventions that promote dual protection of condoms for both contraception and BBVs/STIs.

## Introduction

Blood-borne viruses (BBVs) and sexually transmissible infections (STIs) constitute a global public health threats as they account for annual 2.3 million deaths and 1.2 million cases of cancer [1]. Sexual transmission of BBVs/STIs occurs through unprotected anal, oral and/or vaginal sexual contact between at least two people, one of whom, is infected. International students, who are largely young people, are important population as regards BBVs/STIs prevention and transmission. However, the generic use of the term “international students” in some previous Australian-based studies has led to the recruitment and classification of international students from both conservative and non-conservative sexual cultures into the same participants group [2, 3]. Conflating international students from conservative and non-conservative cultures may result in situations where nuances and complexities around sex-related experiences are lost or, at most, tangentially investigated. Though the two sub-regions of focus in this study––East Asia and sub-Saharan Africa––have diverse sexual cultures, they were targeted partly because they both share largely conservative sexual cultures. These conservative cultures are different from the more liberal sexual cultures which are predominant in Western societies such as Australia [4–6]. Even within the context of conservativeness, it is important to acknowledge there are differences in the sexual cultures of East Asian and sub-Saharan African countries and the sex-related experiences of international students from these countries.

Importantly, the focus of the present study is on sexual transmission of BBVs. Thus, the study does not focus on non-sex-based protective practices such as needle exchange and other harm reduction strategies. Though many strategies provide varying degrees of protection against STIs and the sexual transmission of BBVs, common practices include abstinence, partner fidelity and condom use. These strategies are known as the ABC approach – abstinence, be faithful, correct and consistent condom use [7]. Young people from conservative settings engage in premarital sex, despite the predominance of abstinence expectation of their cultural backgrounds [8]. It is therefore not surprising abstinence-only interventions are ineffective in preventing HIV, STIs and adolescent pregnancy [9, 10]. Of note, migrating to Australia where there are more liberal sexual norms, could influence conservative views and practices toward premarital sexual abstinence. In fact, some international students from conservative cultures believe it is difficult to maintain sexual abstinence in Australia [11]. International students who decide to become sexually active in Australia may have partner fidelity and condom use as possible strategies for protection against BBVs/STIs.

Moreover, mutual fidelity remains a central question and concern in partner fidelity as a protective practice [12, 13]. Thus, correct and consistent condom use within or outside the context of partner fidelity seems to be a more reliable protective practice for sexually active young people. Thus, evidence from Australia [14–16] and elsewhere [17, 18] indicates condom use, consistent or occasional, is a commonly cited BBVs/STIs protection strategy among sexually active young people. However, considering condoms provide dual protection against both pregnancy and BBVs/STIs, it is important to understand the motives for condom use among young people. Understanding condom use motive is vital because of the likelihood of potentially conflicting messages that either stress the prevention of pregnancy over BBVs/STIs or BBVs/STIs over pregnancy [15].

Research evidence among young people in Australia indicates contraception is critical in condom use decisions [14–16]. A large-scale national survey that examined Australian secondary school students’ choices for contraception and STIs prevention showed condom was the choice contraception method among participants [15]. This finding is supported by a more recent study among a sample of predominantly young women in Australia1 [6] and by another study among a general Australian sample [14]. Watson et al. reported the use of dual contraceptive methods; which involve condoms and other contraceptives such as pills [16]. Notably, 1 in 5 of the study participants, who used condoms solely for the purpose of contraception, also reported condomless sex [16]. Thus, when condoms are primarily used for contraception, young people may practise condomless sex, if the chance of pregnancy is low or non-existent. Such instances may include the use of other forms of contraceptives (e.g., emergency contraception), withdrawal, billings ovulation method, and anal/oral sex.

Importantly, understanding BBVs/STIs protective practices among international students is imperative because a large body of evidence links international migration to the global spread of BBVs/STIs [19–21]. Though international university students, with temporary visa status, are not settled migrants; they are considered a migrant population by the United Nations migration agency [22]. Importantly, emerging evidence indicates high proportions of migrants in Western countries who contract HIV, do so postmigration [23–26]. This postmigration HIV acquisition might be attributable to ineffective interventions for migrant populations in high-income countries. To be effective, BBVs/STIs interventions for migrants moving from low or middle-income to high-income countries, must consider the complex interplay between migration experience, health system, and sociocultural factors which influence risk and prevention practices [27].

While Australia has recorded significant progress in HIV prevention and control [28–31], there are concerns around rising notifications of STIs in the general population [32, 33]. There are also concerns around HIV notifications among migrant populations [28, 29, 31]. Specifically, in 2018, 46% of new HIV notifications in Australia occurred among migrants [30]. In addition, while there was a decline in new HIV notification among Australian born residents between 2017 and 2018; there was an increase among migrants, within the same period [30]. The disproportionately higher HIV notification among migrant population in Australia may be linked with high BBVs/STIs related stigma, especially among migrant and international student communities from more conservative cultures. This high stigma may impact prevention, screening and treatment uptake among this population [28, 34].

Notably, international students from backgrounds where BBVs/STIs are stigmatised may experience double vulnerabilities to BBVs/STIs risks. First, international students from more conservative cultures are members of the general migrant population in Australia who are more vulnerable to BBVs/STIs [28–31]. Second, international students are part of the general population of young people who are known to be at higher risk of STIs and the sexual transmission of BBVs [35–37], especially if enrolled in the university [38–41]. The social settings of universities can drive risky sexual practices and increase young people’s vulnerabilities to sexual transmission and acquisition of BBVs/STIs [38, 40, 41]. In fact, some students perceive their time at the university as a period for intensified sexual exploration [39]. Thus, movement of students across international borders may have implications for acquisition and transmission of BBVs/STIs, since transnational mobility is a major driver of the global spread of BBVs/STIs [19–21]. Students who move from sexually conservative backgrounds to a liberal society, such as Australia, may use their migration experience as an opportunity to explore sexual freedoms, thereby initiating or becoming more sexually active [11, 42, 43].

Drawing on Berry’s acculturation model [44, 45], international students from conservative cultures may experience any of the following: *assimilation* (identifying more with host country than home country sexual cultures), *integration* (maintaining both Australian and home sexual cultures), *separation* (rejecting Australian but upholding home sexual cultures) and *marginalization* (rejecting both Australian and home sexual cultures). Of note, these types of acculturation represent a range of possible acculturative experiences international students may have. Therefore, these experiences would differ both in types and levels for different students. Consequently, it may not be feasible to use any of the four experiences (assimilation, integration, separation and/or marginalisation) to represent the acculturative experience of all international students.

On one hand, international students who become more acculturated into liberal sexual cultures may change their sexual practices. This change in sexual practice may occur within the context of inadequate knowledge and other structural factors which impact safer sex practices such as consistent condom use [13, 46, 47]. On the other hand, less acculturated international students may largely network with communities from their home country. Since social isolation is linked to risky sexual practices among international students in Australia [48, 49], gaining social support from communities which share home country norms may reduce BBVs/STIs risks. Moreover, lower sexual acculturation has been associated with higher protective sexual behaviours among migrant adults [50], and young [51–53] populations. Therefore, strong connection with home country communities may enhance maintenance of more traditional sexual norms and practices such as abstinence. Of note, there is paucity of evidence around whether and how acculturation influence BBVs/STIs protective practices among international students who migrate from conservative settings to Australia. Similarly, the role of social support, which has been linked to protective and safer sexual health outcomes among young people [54–56], has not been explored among international students in Sydney.

Furthermore, while sexual health knowledge remains vital in promoting safer sexual practice, it does not always translate into protective practices [57]. Efficacy belief could be a factor in translating sexual health knowledge to protective practices [58]. Thus, while young people may know BBVs/STIs can be prevented through abstinence, partner fidelity or condom use [59–61], self-perceived ability to adopt any of these behaviours is also important. Moreover, though efficacy belief is positively associated with sexual communication [62], talking about sex as a young person may be seen as a taboo in many conservative cultures [2, 11, 42]. This may partly explain the reason why international students who come from conservative cultures have limited sexual health knowledge, efficacy beliefs, and confidence to discuss sexual health concerns or access sexual health services in Australia [2, 3, 48, 49, 63, 64].

Previous studies among samples of international students in Sydney, Australia, who come from more conservative settings, suggest this population may change their more traditional sexual views and practices to more liberal ones, as a result of their contact with sexual norms in Australia [2, 11]. Thus, it is important to understand whether and how any of such changes from traditional sexual views about BBVs/STIs, impact protective sex among sexually active participants. Therefore, it is useful to explore how sexually active international students, who come from conservative backgrounds, understand, practice and rationalise protective sex.

In the Australian context, previous studies have investigated sexual health knowledge, values, norms and risk behaviours among international students, who come from more conservative cultures [2, 3, 11, 13, 42, 46, 48, 49, 63, 64]. However, there is still a research gap on how to strengthen BBVs/STIs protective practices among this population. Thus, in contributing to filling this research gap, quantitative data is used to address the hypothesis international students would be more likely to practice BBVs/STIs protective behaviours if they are: less acculturated to Australian sexual culture, possess greater BBVs/STIs prevention knowledge, have access to sexual health information in Australia, possess greater efficacy belief and have access to emotional social support.

Moreover, beyond understanding predictors of BBVs/STIs protective practices, it is equally important to understand how sexually active international students, who come from conservative backgrounds, perceive and practise protective sex. This way, we would be able to provide more comprehensive and tailored sexual health services which may be effective in meeting the sexual health needs of this population. The purpose of the present study is to understand how to promote primary prevention among international students in Australia, who come from more conservative sexual cultures. Using a mixed-methods design, the present study explores BBVs/STIs protective practices among international university students in Sydney, Australia, who come from East Asian and sub-Saharan African countries and territories. While quantitative data explored predictors of BBVs/STIs protective practices (abstinence, condom use and partner fidelity), qualitative data examined, in greater detail, how sexually active participants understand, practice and rationalise condom use in protective sex.

## Research methods

### Quantitative data

#### Recruitment, sample and sample size estimation

Participants were recruited using electronic and paper flyers. Electronic flyers were advertised in various international student organisations social media pages and included a link to the survey. Targeted recruitment was done focusing international students from East Asian and sub-Saharan African countries, using national student associations. Recruitment was also supported by general international students’ group, although the inclusion criteria were clearly spelt out that only international students from East Asian and sub-Saharan African countries were eligible to participate. In addition, paper flyers with QR code to link the survey were also posted at strategic locations around various university campuses.

A sample size of 300 participants, 150 each from East Asia and sub-Saharan Africa was the original recruitment goal. However, this plan was impacted by the outbreak of COVID-19 pandemic. Participant recruitment was discontinued after enhanced recruitment efforts over a protracted period did not yield desired outcome. Moreover, although over 500 participants accessed the survey link, only 175 participants filled out the survey beyond the first three socio-demographic questions. On further scrutiny for eligibility, responses that were received from IP addresses outside Australia were removed, bringing the sample for analysis to 149. The decision to remove responses received from IP addresses outside Australia was based on the inclusion criterion of surveying only onshore international students. The rationale for using only data provided by onshore international students is because acculturation is a major variable of interest.

The sample size estimation for the quantitative data was informed by the main analytical technique – logistic regression. Therefore, this study employed the Events Per Variable (EPV) sample size estimation method [65]. EPV is widely used in estimating sample sizes for logistic regressions, and is also recognised as a methodological quality index for appraising prediction studies [66, 67]. EPV is relevant in this study because the study uses logistic regressions to examine whether and how the independent variables predict BBVs/STIs protective practices. The EPV method of sample estimation entails having at least 10 participants for each independent variable or predictor variable in a study [66]. However, 20 or more participants per variable have been recommended as a much more adequate number [66] as 10 is considered too conservative [67]. This study uses the more reliable 20 participants per variable to estimate its sample size. Since six predictor variables were fitted into the logistic regression model, the estimated adequate sample size for a valid and reliable prediction is 120 participants. Thus, the sample size of this study is deemed adequate for valid and reliable predictions.

#### Data collection tool and measures

An anonymously distributed online questionnaire was used to collect quantitative data. The questionnaire consisted of items developed using insights from the qualitative data, and an existing acculturation scale originally developed by Ryder and colleagues [68]. While the use of pre-existing validated measures across all the variables in this thesis could enhance data quality as these measures have gone through a rigorous development process [69, 70], the use of such measures among a population they were not validated on could lead to measurement error and conclusions drawn with less confidence [71]. Such measurement error and caveated conclusions are partly because measures arising from validated instrument are not fixed but dependent on context and population [69, 71]. Notably, Australian-based migrants, who come from African and Asian backgrounds, may have difficulties understanding sexual health survey items as intended [72]. Thus, the decision to develop my own measure was informed by a need to use measures that are contextualised to the target groups in this study. This is also in line with my research design––sequential exploratory mixed methods––in which questionnaire items are developed from insights generated from preceding interviews.

The questionnaire draft was reviewed by a team of experts in social psychology, sociology and public health. Thereafter, a draft of the survey instrument was pretested among a sample of Sydney-based international students who come from conservative cultures. This pretesting was partly done to ensure the items of the questionnaire were clear from the perspective of potential participants. Ensuring clarity of the questionnaire items is important because Australian-based migrants, who come from African and Asian backgrounds, may have difficulties understanding sexual health survey items as intended [72]. Feedback from the pilot was used to shorten the length and further improve the wordings of the questionnaire for greater clarity. Reliability test for measures in the questionnaire was conducted using the Cronbach alpha technique. Cronbach alpha scores above 0.70 is considered a strong and acceptable reliability index [73].

The measures in the questionnaire are briefly discussed below:

### Outcome variable

#### BBVs/STIs protective practices

BBVs/STIs protective practices are defined as any strategies intentionally utilised by an individual to prevent the sexual transmission of BBVs/STIs. Common practices such as abstinence, condom use, partner fidelity, PrEP and vaccinations have all been reported as providing varying levels of protection against the transmission of these infections [66, 67]. Participants were asked whether they engaged in the following BBVs/STIs protective behaviours in the last 12 months (1) abstinence (2) consistent condom use, (3) sexual fidelity (having a single partner), (4) occasional condom use (5) other strategies or (6) doing nothing to protect themselves against BBVs/STIs. These responses were categorised in a dichotomous variable of (1) engaging in consistent protective practices (2) engaging in inconsistent or non-protective practices. Participants who were abstinent, practised consistent condom use or practised partner fidelity (or alongside consistent/occasional condom use) were classified as engaging in high protective practices. Participants who reported occasional condom use (without practising partner fidelity) and those who reported not doing anything in the past 12 months to protect themselves against BBVs/STIs (but not abstinent) were all categorised as not engaging in protective practices.

### Explanatory variables

- BBVs/STIs prevention knowledge:

This part of the survey consisted of 11 items which assessed participants’ knowledge about BBVs/STIs prevention. These prevention knowledge items (e.g., BBVs/STIs cannot be transmitted through oral sex) were informed by insights generated from the first phase of qualitative data. The items have three response options: “True”, “False”, and “I do not know.” Items in this section were scaled so higher scores indicate greater BBVs/STIs prevention knowledge. In scaling the items, correct responses were scored 2, incorrect responses were scored 1 and “I do not know” responses were scored 0. No reliability analysis was undertaken for this section as Cronbach Alpha is not appropriate for a knowledge-based scale [69].

- Sexual acculturation scale:

Participants were asked questions about the way they believed their sexual practice is influenced by sexual norms in Australia or sexual norms in their home country. This scale was modified from the Vancouver Index of Acculturation, originally developed by Ryder et al. [68], using insights from the qualitative interviews. The scale has a total of eight items which were summed up, with higher scores indicative of greater acculturation to Australian culture (i.e., higher levels of adopting or identifying more with Australian sexual culture). The scale yielded 0.85 reliability on the Cronbach alpha index.

- Sexual health information access scale:

This scale comprised three items designed to generate data about participants’ access to sexual health information in Australia. Items in this section (e.g., I have access to sexual health information in Australia) were developed from insights from the qualitative data. The response options were on a five-point rating scale ranging from “True for me” to “Untrue for me”. The section was scaled by summing the items so higher scores indicate greater access to sexual health information and vice-versa. This scale yielded a reliability index of 0.74 on the Cronbach alpha scale.

- Emotional support scale:

This section contains 4 items designed to generate data on participants’ access to emotional social support. Items in this section were developed from insights gained from the qualitative interviews. Response options were on a five-point scale ranging from “True for me” to “Untrue for me.” Items in this section of the questionnaire (e.g., I have someone to talk to when I need advice) were scaled by summation so higher scores represent greater access to emotional support. The reliability of this scale (α = 0.83) was estimated using the Cronbach alpha method.

- Conservative sexual norm scale:

This scale comprised eleven items which were developed from insights generated from the qualitative interviews. Items in this scale focused on abstinence expectation, gender norms, sexual communications, and stigma beliefs around blood-borne viruses and STIs. The items have five points response options ranging from “Strongly agree” to “Strongly disagree.” These items were scaled by summing the items so higher score indicates higher levels of conservative norms. The scale yielded a reliability index of 0.96 on the Cronbach alpha scale.

- Efficacy belief scale:

This scale was developed to generate data about participants’ BBVs/STIs protection efficacy belief. The section consisted of three items (e.g., I can handle unforeseen sexual situations) with five points responses ranging from “Always” to “Never”. The items in this section of the questionnaire were developed from insights from the qualitative data. These items were scaled by summing the items so higher scores indicate greater efficacy belief. The scale has a reliability index of 0.73 on the Cronbach alpha scale.

- Sample characteristics

Demographic characteristics included age, gender identity, sexual identity, background. Participants were also asked about their monthly income in Australian dollars, the amount of time they had spent in Australia and their marital status. Data about sexual practices were also collected on age at first sexual experience (if sexually active), number of sexual partners ever had in Australia and number of sexual partners in the last 12 months.

#### Data collection

An information and consent sheet preceded the questionnaire, which was administered electronically via UNSW Qualtrics. Potential participants provided informed consent by clicking on the appropriate consent page before being directed to the questionnaire. Data collection occurred over an extended period (between November 2019–March 2021) due to the impact of the COVID-19 pandemic. The questionnaire was electronically and anonymously completed as no identifying personal information was collected. Ethical clearance (HC190215) was provided by the UNSW Human Research Ethics Committee. Participants’ compensation involved a prize draw (1 in 20) of 100 AUD gift voucher. Identifying data provided by participants, who indicated interested to enter the prize draw for compensation, was stored separately from provided responses to the questionnaire.

#### Data analysis

A bivariate analysis using Spearman correlation was conducted to assess the relationship between the outcome and explanatory variables. A binary logistic regression analysis was conducted to model participants’ likelihood to engage in BBVs/STIs protective practice by age, BBVs/STIs prevention knowledge, acculturation, access to BBVs/STIs information in Australia, emotional support and conservative sexual norms. Efficacy belief was not included in logistic regression model because the relationship between efficacy belief and the outcome variable was not significant. In addition, gender and region were not included in the regression model because preliminary chi-square tests showed there is no significant difference in protective sexual practices by these two variables. All tests were conducted using the 0.01 alpha level to indicate significance. Listwise deletion method was used to handle missing data. Thus, any entry with a missing value in at least one of the specified variables, was dropped from the analysis. Statistical analysis was performed using SPSS version 27.

### Qualitative data methods

The methods for the qualitative data have been explained in greater details elsewhere [11]. Qualitative data was provided by 20 participants through semi-structured in-depth interviews, which were conducted between May and August 2019. Based on participants’ preferences, the interviews were conducted face-to-face or over the telephone. At the interviews, participants discussed about their BBVs/STIs protective practices, and about the practices of their friends and other international students, from more conservative cultures. Participants received a $40 gift voucher as reimbursement for possible costs associated with participating in the interviews. The interviews lasted between 40 and 85 minutes, and were conducted in English. Before each interview, participants provided either a written (face-to-face) or verbal (telephone) informed consent.

All the interview sessions were audio-recorded and transcribed verbatim for analysis. This study adopted the reflexive thematic analysis (RTA) method, which emphasizes the importance of researchers’ subjectivity as analytic resource [74, 75]. For familiarisation and immersion, data transcripts and field notes were iteratively read multiple times; while listening to the audio recording of the interviews. The transcripts were thereafter coded by the researcher using QSR NVivo (12.6.0). It is important to stress while multiple coding is possible in RTA, the essence of such multiple coding is neither for consensus nor agreement [75]. The coding approach in the present study combined inductive and deductive strategies, thereby grounding the codes on both generated data and perspectives from empirical literature [75]. Themes were generated through combining, reviewing and refining the codes.

## Results

### Quantitative data findings

#### Socio-demographic characteristics

In the survey sample as shown in Table 1, participants were almost evenly distributed across the three age categories with adolescents and young adults (18–24 years) accounting for 32.2%. Most participants identified as heterosexuals (85.2%) and were never married (63.8%). The largest proportion of the participants (38.3%) had spent between 13 and 24 months in Australia. Further, the largest proportion of the participants (32.2%) reported having their first sexual experience in their teens. Similarly, the largest proportion (33.6%) reported between 1 and 3 sexual partners in Australia. Self-reported BBVs/STIs protective practices in the last 12 months included abstinence (28.7%), consistent condom use (19.9%), occasional condom use (18.7%) and having a single partner (25.1%) (Table 1). Of note, condom use (consistent and occasional use) seems to be the commonly reported protective strategy among sexually active participants (38.6%), hence my motivation to go back to the previously collected qualitative data set to explore how condom use is enacted as presented in subsequent section.

**Table 1:** Sample characteristics

Variables	N (%)
**Gender**	
Man/male	72 (48.3)
Woman/female	72 (48.3)
Non-binary	5 (3.4)
**Age (in years)**	
18–24	48 (32.2)
25–29	46 (30.9)
30 and above	55 (36.9)
**Sexual Orientation**	
Gay/homosexual	7 (4.7)
Bisexual/pansexual	4 (2.7)
Heterosexual/straight	127 (85.2)
Lesbian	6 (4.0)
Queer	1 (0.7)
Different term/prefer not to say	4 (2.7)
**Background**	
East Asia	80 (53.7)
Sub-Saharan Africa	69 (46.3)
**Time spent in Australia to date**	
3–12 months	41 (27.5)
13–24 months	57 (38.3)
25 months or more	51 (34.2)
**Monthly income (living expenses)**	
Less than $1000	26 (17.4)
$1000–$2000	38 (25.5)
$3000 or more	85 (57.0)
**Marital status**	
Single	95 (63.8)
Spouse or partner overseas	22 (14.8)
Living with spouse/partner	29 (19.5)
Divorced	3 (2.0)
**Age at first sex (in years)**	
Never had sex	23 (17.8)
Below 15	14 (10.9)
15–19	48 (37.2)
20–24	34 (26.4)
25–29	6 (4.7)
30 and above	4 (3.1)
**Sexual history in Australia**	
None (abstinent)	44 (34.1)
1–3	50 (38.8)
4–6	21 (16.3)
7 and above	14 (10.8)
**BBVs/STIs protective practices in last 12 months**	
Abstinence	49 (28.7)
Consistent condom use	34 (19.9)
Occasional condom use	32 (18.7)
Single partner	43 (25.1)
Other strategies	3 (1.8)
Nothing	10 (5.8)

#### Bivariate analysis

Spearman rho correlation analysis was used to assess the relationship between each of the explanatory variables and the outcome variable (Table 2). The results of this bivariate analysis show higher BBVs/STIs prevention knowledge (*r* = 0.258, *p* < 0.01), lower acculturation into Australian sexual culture (*r* = − 0.395, *p* < 0.01), and greater emotional social support (*r* = 0.311, *p* < 0.01) were significantly associated with increased protective practices. Similarly, greater access to sexual health information (*r* = 0.257, *p* < 0.01), lesser conservative sexual norms (*r* = − 0.265, *p* < 0.01) and older age (*r* = 0.325, *p* < 0.01) were also significantly associated with greater practice of protective behaviours. However, no significant relationship was found between efficacy belief and protective practices (*r* = 0.159, *p* > 0.05); even though a positive relationship exists between these variables. To establish the variables that remain independent predictors of BBVs/STIs protective practices, all variables significant at the bivariate level were entered into a logistic regression model.

**Table 2:** Spearman correlations among outcome and explanatory variables

	^c^1^b^	2	3	4	5	6	7	8
^b^BBVs/STIs Protective Practice [1]		.325^a^	.258^a^	-.395^a^	.311^a^	.257^a^	-.265^a^	.159
Age [2]			.358^a^	-.231^a^	.268^a^	.352^a^	-.254^a^	.179^*^
BBVs/STIs Prevention Knowledge [3]				-.250^a^	.346^a^	.460^a^	−.105	.326^a^
Sexual Acculturation [4]					-.282^a^	−.154	.206^*^	−.079
Emotional Social Support [5]						.463^a^	-.241^a^	.416^a^
Access to Sexual Health Information [6]							−.088	.419^a^
Conservative Sexual Norms [7]								.039
Efficacy Belief [8]								

^a^Correlation is significant at the 0.01 level (2-tailed)

^b^Outcome variable

^c^The numbers in the column correspond with the numbers in the square brackets in the row, indicating the appropriate variables

#### Logistic regression: predictive effects of explanatory variables on outcome variables

A logistic regression was performed to examine the effects of BBVs/STIs prevention knowledge, acculturation, emotional support, access to sexual health information, conservative sexual norms and age on the likelihood participants engaged in BBVs/STIs protective practices. The logistic regression model was statistically significant, *X*^2^(6) = 31.33, *p* < 0.01 and the model explained 33.1% (Nagelkerke *R*^2^) of the variance in engaging in BBVs/STIs protective practices (Table 3). However, only acculturation contributed significantly to the model (OR = 0.833, 95% CI = 0.820–0.952). The findings from this study indicate participants with greater level of acculturation were less likely to engage in BBVs/STIs protective practices. This is because of the lower odds (OR < 1) of association between acculturation and BBVs/STIs protective practices (Table 3). In addition, while there is greater odd of association (OR > 1) between other explanatory variables and BBVs/STIs protective practices, such associations are not statistically significant as the 95% CI include 1 for all these variables (Table 3).

**Table 3:** Logistic regression table of BBVs/STIs protective practice and explanatory variables

Explanatory Variables	Odds Ratio	95% CI
BBVs/STIs Prevention Knowledge	1.016	0.911–1.132
Sexual Acculturation	.883	0.820–0.952
Emotional Social Support	1.030	0.887–1.196
Access to Sexual Health Information	1.069	0.879–1.300
Conservative Sexual Norms	1.006	0.981–1.032
Age	1.096	1.002–1.199
Constant	.938	

Model *X*^2^ = 31.33, *p* < 0.01, *R*^2^ = .333

### Qualitative results

#### Demographic characteristics

Most participants were aged 18–31 years (16/20), were never married (16/20), and identified as females (11/20). Only one of the participants, who is from East Asia, identified as LGBTQI+. Half of the participants were from East Asian countries and territories, while the other half were from sub-Saharan African countries.

#### Themes generated on condom use practices

As stated earlier, findings from the quantitative data indicated condom use (consistent or occasional) was the most commonly cited BBVs/STIs protective practice. Therefore, interview data was used to further explore condom use motives and practices among a sample of the study population who are sexually active. This study generated two themes regarding condom use practices in protective sex among sexually active participants: (1) prioritising contraception over BBVs/STIs prevention and (2) acculturation-related logic for underestimating BBVs/STIs risks.

##### Contraception is prioritised over preventing BBVs/STIs in condom use

Experiences shared by some sexually active participants indicate condom use is primarily motivated by contraception. The interview data suggest the need to conceal being sexually active partly contributes to making contraception imperative. Concealing sexual activities may be necessitated by predominant premarital abstinence norm in the conservative sexual cultures of most East Asian and sub-Saharan African societies.

Of note, when specifically asked about BBVs/STIs protective practices, almost all the sexually active participants mentioned consistent or occasional condom use. However, they also disclosed the main reason for using a condom is to prevent pregnancy, with BBVs/STIs described as a secondary concern:

*Just condoms, I don’t want to get pregnant … My greatest motivation is [the] fear of my parents because I know my parents. If I tell them I am pregnant, you know my parents are African parents … I am too scared. It [BBVs/STIs] is the second thing on the list; after pregnancy, it’s STIs* (female/19/sub-Saharan Africa).

While some participants may view BBVs/STIs as secondary in their condom use practices, others may not even consider preventing BBVs/STIs as an issue of concern. Perspectives from the interviews indicate contraception is prioritised over BBVs/STIs prevention in both stable relationships and casual hook ups. As mentioned earlier, one common reason for acting to prevent pregnancy is to conceal being sexually active, as another participant clearly pointed out:

*It [using condom] is about birth control; not about STIs … I always use condoms … [because] if our parents know … they will be very angry because in [home country] people are not supposed to have sex before [marriage]* (male/26/East Asia).

Furthermore, the importance of contraception was also attributed to career goals and aspirations. According to some participants’ perspectives, unplanned pregnancy could disrupt career plans and aspirations. One of the participants described this motivation while sharing her experience:

*I don’t want a baby before a proper age … some of my friends are having child [ren] now … I can’t say their lives will be ruined, but they [will] need to spend most of their time to take care of the children … I think of maybe 20[s] [to] be a good age for [pursuing] career [goals] … So, I just don’t want to have a baby so early … I want to take one more degree in the future, maybe an MBA in UK or US. So, if I have a baby now, then I can’t pursue this dream* (female/22/East Asia).

Moreover, the need to prevent pregnancy may have led some participants to rethink safe or protected sex as pregnancy prevention. During the interviews, some participants described a *safe* or *protected* sexual practice as not involving the risk of pregnancy. Thus, sex may be seen as protected or safe only when pregnancy is prevented:

*We used a condom because I don’t want to get pregnant … for me, protection is number one because I don’t want to get pregnant* (female/19/East Asia).

*… safe sex, you know, to prevent being pregnant* (Male/22/East Asia).

Similarly, while responding to interview questions around BBVs/STIs protective practices, other participants mentioned the use of contraceptive pills only, without condoms. Though contraceptive pills prevent pregnancy, they do not provide protection against BBVs/STIs if pills are not used together with condoms for dual protection.

*It [sex] was protected, except it wasn’t [with] a condom, but I take birth control pills. So, technically, essentially, it’s still protection* (female/19/SSA).

Moreover, the centrality of preventing pregnancy in condom use may elevate BBVs/STIs risk. This is because individuals may decide to adopt sexual practices which prevent pregnancy, even if such practices elevate the risk of BBVs/STIs. For instance, a participant narrated an anecdote of a personal experience involving condomless anal sex with a casual partner. Anal sex became an alternative to prevent pregnancy since they had no condom on them:

*I didn’t want him to stick anything [penis] in my vagina … So, [I] went for that [anal sex].*

Interviewer: Okay. Was it because there was no condom that you didn’t want to go vaginal?

*Yeah. Actually, that was my main reason. Because my friend and I always had this conversation, even if he says his pull-out game [withdrawal] is strong … you can’t trust [that] because, a little bit [sperm] can go in and then you [will] have to deal with that*.

Interviewer: Okay, your greatest [concern] is just pregnancy, not BBVs/STIs?

*For me, yeah* (female/19/sub-Saharan Africa).

##### Acculturation-related logic for underestimating BBVs/STIs risks

The condom use practices of some interview participants, who are sexually active, clearly suggest these participants may be underplaying the risks of BBVs/STIs and focusing greater attention on preventing pregnancy. Thus, while acting to prevent pregnancy, the risks for BBVs/STIs may be ignored or even elevated.

Since Australia may have a lower prevalence of BBVs/STIs compared to participants’ home countries, Australia may be seen as a setting with low or no risk for BBVs/STIs infection. Thus, in line with findings from the quantitative data, greater acculturation into the Australian society––where BBVs/STIs are less of a concern because of low prevalence––may encourage low BBVs/STIs protection among some sexually active participants. This logic of feeling less concerned with BBVs/STIs risks in Australia may be a group thinking logic as suggested by the account of one participant:

*… when partying and drinking with my friends, we talk [about] a lot of topics … we talk about STIs, like it is highly impossible in Australia … you know, it’s very rare here* (male/22/East Asia).

Furthermore, the interviews data suggest some sexually active participants believe there is available treatment for BBV/STIs in Australia and therefore dismiss the risk associated with these infections. One participant stressed this logic in justifying her reasons for using condoms primarily to prevent pregnancy:

*… [there’s] nothing wrong to have it [BBVs/STIs] because nowadays, there are so many medicines and treatment … nowadays STDs [BBVs/STIs] is not curable, but it’s treatable* (female/19/East Asia).

Similarly, perspectives shared in the interviews indicate since incurable BBVs/STIs such as HIV are manageable and under control, these infections may not affect the natural life span:

… *this is my understanding that even HIV is under control. So, if you get HIV, if you get proper treatment, you can live till your natural end of life, and yeah, so it’s manageable … apart from HIV, any STIs are curable* (male/39/East Asia).

However, though BBVs/STIs are curable or treatable, there may be concerns around engagement with sexual health services among this group of international students. During one of the interview sessions, a participant linked her hesitation to seek BBVs/STIs screening and/or treatment to perceived or anticipated stigma:

*Stigmatisation is a thing, very much a thing … because if you say, “I’m going to go get checked for STIs,” … “Oh, so you’ve been sexually active?” So, you’re already feeling judged … even when you go to the clinic itself, as a minority, you feel a sense of, “I’m not going to get quality service … they are not going to want to help me.” So, you just start saying, “You know what, maybe there’s no point in me going” and just pray that I don’t get STIs* (female/19/sub-Saharan Africa).

## Discussion

This mixed-methods study explored BBVs/STIs protective practices among international students in Australia, who come from East Asia and sub-Saharan Africa which are more conservative cultures. While the quantitative data explored predictors of BBVs/STIs protective practices, the qualitative data closely examined condom-use motives and practices among another sample of this group of international students. The quantitative findings from this study showed consistent or occasional condom use as the commonly cited BBVs/STIs prevention strategy. However, the qualitative results revealed that just as other young people [15, 17, 18], this group of international students may be more motivated by contraception over BBVs/STIs prevention in their condom use practices; which may have implications for BBVs/STIs risks.

Consistent with previous findings linking less acculturation with protective sexual practices [50–53], participants in the present study, who reported lesser acculturation were likely to report greater BBVs/STIs protective practices. This suggests international students from conservative cultures with greater acculturation may be less likely to practice BBVs/STIs protective behaviours. Of note, international students in Australia, who come from conservative sexual backgrounds may hold a normative view of casual and permissive sex among young people in Australia [11, 42, 76]. Moreover, previous Australian evidence shows domestic students engage in lesser protective practices than international students [63, 64]. Thus, international students from more conservative backgrounds, who become more acculturated to real or perceived sexual norms in Australia, may likely practice lesser BBVs/STIs protective behaviours. Conversely, less acculturated international students may be more likely to practise sexual abstinence and/or partner fidelity, partly due to the maintenance of the more conservative and restrictive sexual norms of their backgrounds.

This pattern of finding is also seen in the qualitative data, which suggests greater acculturation––to the more liberal norms in Australia––may influence sexually active international students to underestimate the risks of BBVs/STIs in Australia. BBVs/STIs are largely stigmatised in conservative cultures and among migrant communities in Western societies, who come from such conservative cultures [34]. However, evidence among international students in Sydney, who come from such cultures suggests this group of international students change some of their conservative sexual views and practices as they become acculturated into the Australian society [2, 11]. Therefore, the qualitative results of this study, in support of these previous studies, suggest with acculturation, this group of international students may adopt more liberal views towards BBVs/STIs. The belief BBVs/STIs treatment is available and accessible in Australia, may be contributing to these more liberal sexual views and practices. Consequently, holding more liberal sexual views may also influence this group of international students to disregard or underestimate the risks of BBVs/STIs.

In this present study, qualitative data results unpack the quantitative data results which link both greater acculturation, and conservative norms to less BBVs/STIs protective practices. Previous Australian studies have suggested contraception plays a key role in young people’s condom use practices [14, 15]. On the other hand, international students in Australia, who come from more conservative cultures, may change their conservative sexual views and practices [2, 11, 42, 43]. This change in sexual views and practices may also co-exist with maintaining some conservative beliefs, which may impact BBVs/STIs prevention [3, 77]. For instance, unplanned pregnancies among unmarried young people carry negative social stigma [78], and this stigma may be greater in more conservative cultures because of premarital abstinence expectations.

Thus, both acculturation and conservative norms around premarital abstinence may intersect to influence condom use motives among this group of international students. As regards acculturation, consistent with evidence among young people in Australia [15], and Scotland [17], contraception may be a key motive in the condom use practices of sexually active East Asian and sub-Saharan African international students. As regards conservative norms about premarital abstinence, to prevent pregnancy, the risks of BBVs/STIs may be ignored or even elevated as demonstrated in reported practices such as withdrawal, and condomless anal sex. Consequently, this group of international students may benefit from condom promotion interventions which focus on dual protection to promote the prevention of both unplanned pregnancy and BBVs/STIs.

In addition, both acculturation and conservative norms could also impact abstinence and partner fidelity as protective practices. Both abstinence and partner fidelity could be described as indicators of the more restrictive conservative norms around young people’s sexuality. Therefore, with greater acculturation to the more liberal and less restrictive sexual norms in Western societies [5, 6, 11], abstinence and partner fidelity may become weakened, while casual sex may become more attractive [11, 13].

In support of previous studies among samples of migrant populations in Australia [76] and Canada [77], some of the participants in the present study may hold the view BBVs/STIs are rare in Australia. This belief, may be common among more acculturated participants, considering most young people in Australia have low STIs risk perception [79]. Therefore, it is likely more acculturated sexually active international students may view Australia as a safe setting; where BBVs/STIs risks are either low or non-existent. As such, they may be less likely to engage in practices which are protective against BBVs/STIs, as the quantitative and qualitative results of this study indicate. This logic of downplaying BBVs/STIs risks in low prevalence countries may explain HIV acquisition; when people migrate from high prevalence settings such as sub-Saharan African countries to low prevalence countries such as UK [25], France [23, 24], European countries [80–82], and high-income countries generally [26].

In addition, in support of previous studies [83–85], the results from the qualitative data in this study suggest BBVs/STIs treatment could constitute risk-compensation, which sexually active participants may use to rationalise less BBVs/STIs protective practices. This may be more likely among acculturated international students; whose more liberal views could involve underestimating BBVs/STIs threats because of the availability of cure/treatment. Although incurable BBVs such as HIV can be effectively managed through treatment, until recently, international students––being ineligible for Medicare––did not have access to free HIV treatment. While HIV treatment may become available to everyone in Australia, despite Medicare status, international students from more conservative backgrounds may lack requisite social skills to navigate sexual health services in Australia [3, 42].

International students may also feel a sense of anticipated stigma and may have less trust in health services because these services may be perceived to be culturally insensitive [86, 87]. This sense of stigma as well as low trust, could impact their engagement with screening and treatment sexual health services, despite availability. Thus, migrant populations are more likely to present late for HIV treatment in Australia [29], and other Western societies such as US [88], Europe [89] and other high-income countries [26]. Moreover, even if international students access HIV treatment in Australia, continuing such treatments on return to their home countries, after studying in Australia may be impacted by availability, accessibility and other structural issues [90, 91]. Therefore, primary prevention is imperative for this group of international students.

The quantitative findings from this study also show that older participants are more likely to practise protective sexual behaviours than younger ones, and this may be attributable to some reasons. Younger international students are likely to assimilate a new society’s way of life because they are likely to be more impressionable than older ones [92, 93]. Therefore, younger international students may be experiencing greater acculturation to Australian more liberal sexual attitudes. Besides, older participants may have come to Australia with family members such as spouses or have a spouse in their home country to whom they may remain committed. Older participants may also be more likely to integrate into communities similar to their home community in the new environment and as such, may remain connected to conservative cultural norms. In contrast, younger participants who may have lesser social supports from the sources mentioned earlier, may experience higher levels of social isolation which has been associated with less protective sexual practices among international students in Australia [48, 49]. Further, older participants may also practise more greater protective behaviours because they may be more knowledgeable [79] or may have greater sexual experience than younger participants [94]. Though evidence on the influence of age on sexual practice among young people is mixed [94, 95], the results of the present study support evidence linking older age to protective sexual practices [79, 94]. Thus, younger students, may be a sub-group of interest in interventions aimed at improving BBVs/STIs protective practices among East Asian and sub-Saharan African international students.

However, though not independent predictors, the quantitative results of this study, show that participants with higher BBVs/STIs prevention knowledge, greater emotional support, and greater access to sexual health information, were more likely to report BBVs/STIs protective practices. International students in Australia, who come from conservative sexual cultures have low sexual health knowledge [49, 63, 64], and experience difficulties accessing sexual health services [48, 49]. Therefore, the results of this study highlight a need for tailored sexual health services and social support to engage this group of international students with sexual health services in Australia. Although efficacy belief has been found to predict protective sexual behaviours [58, 79], this was not found in the present study. However, given the results of the present study showed a positive relationship between efficacy belief and BBVs/STIs protective practices, improving efficacy beliefs may also be an important empowerment strategy for safer sexual practices among the population of this study.

Beyond conventional sexual health programs, population-specific and tailored interventions are advocated for international students in Australia, who come from conservative cultures. Such interventions need to combine a range of strategies to improve social support and enhance effective integration of this group of international students into societies with more liberal sexual norms. The essence of effective integration is to modify misconceptions young people from conservative backgrounds may have about sexual practices and BBVs/STIs risks in Western societies. In the same vein, well-integrated migrants are less likely to experience migration-related distress and/or acculturative stress [96, 97], which has been linked to poorer sexual and reproductive health outcomes among international students in Australia [48, 49]. Equally important, delivering such interventions in non-clinical, and peer-supportive social environment is important for wider reach [98, 99], and for effectiveness [99].

### Limitations and suggestions for future studies

The results of this study need to be understood within some caveats. First, the study is cross-sectional and hence causality cannot be established. Second, non-representativeness because of the voluntary nature of recruitment, may also impact generalising the study results to all international students from conservative sexual cultures. Third, considering sex is a subject treated discreetly, and given the traditional cultural background of participants, it is not unlikely some participants may provide socially desirable responses. However, distributing the survey anonymously mitigated against the social desirability bias. Of note, since the survey was electronically administered, it is not unlikely that individuals who may not be part of the population could have access to the recruitment link.

Moreover, this study did not examine the impact of socio-economic, and peer interaction factors on BBVs/STIs protective practices. Future studies could explore these variables as they may also influence BBVs/STIs protective practices of international students. In addition, future studies with potentially larger sample size, could examine how the explanatory variables in this study predict abstinence, partner fidelity and consistent condom use as separate protective practices. Such investigation could not be done in the present study because of limited sample size.

Furthermore, this thesis did not use pre-existing measures; which are deemed useful because they are rigorously developed [69]. However, the usefulness of such pre-existing measures is context- and population-dependent [69, 71]. For context, this thesis used measures that were developed from perspectives shared by the study population; in order to increase the reliability and validity of the conclusions drawn about the sample [71]. Nonetheless, it is suggested that future studies consider selecting or adapting appropriate pre-existing measures. Results from such pre-existing measures could be compared with the results from the present study that used measures developed from the perspectives of population of international students from more conservative cultures. Equally important, future researchers may need to consider the “Table 2 fallacy” [100] especially if establishing causality is intended. Finally, quantitative data collection occurred during the COVID-19 pandemic lockdown, and this lockdown may have impacted participants’ sexual practices. The impact of the pandemic on BBVs/STIs protective practices among a sample drawn from the population of this study has been discussed elsewhere [101].

## Conclusion

This study revealed that less acculturated East Asian and sub-Saharan African international students are more likely to practise BBVs/STIs protective behaviours than more acculturated ones. However, BBVs/STIs protective practices among sexually active participants may be complex because of the primacy of contraception over BBVs/STIs. These findings suggest a need for population-specific interventions aimed at addressing a range of factors associated with BBVs/STIs protective practices. In addition, effective promotion of dual protection in condom use practices among this group of international students is recommended.

## Data Availability

The datasets used and/or analysed in this study will be provided by the author upon a reasonable request.

## Abbreviations

AIDS: Acquired immunodeficiency syndrome
ART: Anti-retroviral therapy
BBVs/STIs: Blood-borne viruses/sexually transmissible infections
EPV: Events per variable
HBV: Hepatitis B virus
HIV: Human immunodeficiency virus
HPV: Human papilloma virus
HSV: Herpes simplex virus
PrEP: Preexposure prophylaxis
RTA: Reflexive thematic analysis
